# Soluble HLA-G (sHLA-G) measurement might be useful as an early diagnostic biomarker and screening test for gastric cancer

**DOI:** 10.1038/s41598-023-40132-6

**Published:** 2023-08-12

**Authors:** Lidy Vannessa Mejía-Guarnizo, Paula Stefanny Monroy-Camacho, Daniel Esteban Rincón-Rodríguez, Andrés Rincón-Riveros, Daniel Augusto Martinez-Vargas, Carlos Alexander Huertas-Caro, Ricardo Oliveros-Wilches, Ricardo Sanchez-Pedraza, Marcela Nuñez-Lemus, Carlos Felipe Cristancho-Lievano, Adriana Milena Castellanos-Moreno, Lina María Martinez-Correa, Josefa Antonia Rodríguez-García

**Affiliations:** 1 Cancer Biology Research Group, Instituto Nacional de Cancerología, Bogotá, Colombia; 2 Research Support and Monitoring Group, Instituto Nacional de Cancerología, Bogotá, Colombia; 3 Gastroenterology Unit, Instituto Nacional de Cancerología, Bogotá, Colombia; 4 Research Group REMA, Universidad Colegio Mayor de Cundinamarca, Bogotá, Colombia

**Keywords:** Cancer, Immunology, Molecular biology, Gastroenterology, Oncology

## Abstract

Gastric cancer (GC) is the fifth most frequent malignancy worldwide and has a high mortality rate related to late diagnosis. Although the gold standard for the GC diagnosis is endoscopy with biopsy, nonetheless, it is not cost-effective and is invasive for the patient. The Human leukocyte antigen G (HLA-G) molecule is a checkpoint of the immune response. Its overexpression in cancer is associated with immune evasion, metastasis, poor prognosis, and lower overall survival. We evaluate the plasma levels of soluble HLA-G, (sHLA-G) in patients with GC and benign gastric pathologies using an ELISA test. A higher concentration of sHLA-G in patients with GC than in those with benign pathologies, higher levels of plasma sHLA-G in women with GC compared with men and significant differences in the sHLA-G levels between the benign gastric pathologies evaluated, was our main findings. As no significant differences were found between the GC assessed stages in our study population, we suggest that sHLA-G is not an adequate marker for staging GC, but it does have diagnostic potential. In addition to providing information on the potential of sHLA-G as a diagnostic marker for GC, our study demonstrate that HLA-G molecules can be found in the membrane of exosomes, which highlights the need to perform studies with a larger number of samples to explore the functional implications of HLA-G positive exosomes in the context of gastric cancer, and to determine the clinical significance and possible applications of these findings in the development of non-invasive diagnostic methods.

## Introduction

The International Agency for Research on Cancer (IARC) in 2020 reports a significant rise in the annual burden of GC, with an estimated 1.8 million new cases and approximately 1.3 million deaths expected by 2040, representing alarming increases of approximately 63% and 66%, respectively^1^. GC incidence and mortality rates are comparable across most of the Americas attributed to the lack of screening measures and ineffective therapeutic strategies, particularly for the advanced stages of the disease. While endoscopy with biopsy remains the most reliable diagnostic tool for GC, its invasiveness and associated costs make it less suitable for routine screening purposes^2^. The use of a non-invasive and easily accessible sample, such as liquid biopsy, which includes samples like blood, saliva, urine, and others^3,4^, coupled with a simple and inexpensive test such as ELISA, for detecting tumor-released molecules would significantly contribute to early detection efforts and potentially save lives by identifying gastric cancer at its earliest stages.

Currently, several tumor markers such as: alpha-fetoprotein (AFP), and carbohydrate antigen 72-4 (CA72-4) can be detected in serum, and are available for clinical applications, since they have a high diagnostic sensitivity and specificity for several types of digestive tract, lung, and ovarian cancers^5^. However, these markers do not have enough sensitivity and specificity for the GC diagnosis^6^.

HLA-G is a non-classical HLA class I molecule whose principal function is tolerogenic, inhibiting all the immune system cells. This molecule has a dynamic expression pattern determined by the presence of seven isoforms generated by alternative splicing of the primary transcript: four are membrane-anchored (HLA-G1-G4) and three are soluble and secreted (sHLA-G5-G7). Additionally, another soluble molecule is generated by proteolytic cleavage of the membrane HLA-G1 isoform [membrane HLA-G1 isoform^7,8^. It has been reported that HLA-G can be found in the tumor cell membrane, free in plasma, or as a membrane bond molecule on tumor cells-derived exosomes. Considering the impact of the immunoregulation induced by HLA-G expression and considering the role that different HLA-G subcomponents can have in the immune system of cancer patients, it can be assumed that the HLA-G detection in any of its forms may have clinical utility as a biomarker for the GC screening, in patients with gastric symptoms, to support the early diagnosis and follow up. Moreover, due to the wide distribution of HLA-G receptors on all the immune cells, HLA-G could have the potential as a therapeutic target for the control of several cancer types.

In GC, HLA-G expression correlates with a Treg cell infiltration associated with tumor progression and a low 5-year overall survival rate. This immune checkpoint molecule participates in immune evasion inducing an increased frequency of Treg cell infiltrates, suggesting that its expression may be a good biomarker of a worse prognosis and a therapeutic target for immunotherapy in GC^9^. On the other hand, there is enough evidence that the HLA-G expression can be useful for the differential diagnosis between healthy controls, benign gastric diseases, and malignancies such as gastric, colon, esophageal, and lung cancer, and thus, it may be a useful biomarker for cancer screening^10^. HLA-G expression is associated with clinical parameters such as disease stage, differentiation degree and nodular status, and contribute to create an immune tolerant microenvironment, allowing the tumor to escape immunosurveillance^11^. It has been demonstrated that the incorporation of sHLA-G and other relevant cancer biomarkers such as CA125, CA19-9 and CA72-4, as a combined diagnostic tool, improves the precision and reliability of GC screening and diagnosis, contributing to better patient outcomes^12^. However, prospective studies are required to validate the sHLA-G potential as a serum biomarker for diagnosis, prognosis, or treatment response^13^.

sHLA-G level can be considered also as a useful indicator for the early diagnosis because high sHLA-G expression levels are associated with early stages in GC patients and induces immune suppression, facilitating tumor progression^14,15^. Additionally, different sHLA-G subcomponents may represent dissimilar qualitative prognostic impacts on the clinical outcome as sHLA-G expression in exosomes (sHLA-Gev) is associated with disease progression and high free sHLA-G is associated with longer overall progression-free survival^16^, and considering that the diagnostic potential of HLA-G in cancer has not been explored in Colombia, we evaluated the plasma levels of free sHLA-G in patients with benign gastric pathology and GC using an ELISA assay, to determine if the expression levels of this molecule in plasma can be used for the early detection of GC and to predict the clinical course of the disease in these patients.

## Results

### Characteristics of the population

sHLA-G Plasma levels were quantified on N = 480 patients: 36.1% (n = 173) with GC and the remaining 63.9% (n = 307) with benign gastric pathology. Demographic and clinical characteristics are presented in Tables 1 and 2.

**Table 1:** Demographic and clinical characteristics of patients diagnosed with gastric cancer.

Characteristic	N = 173	sHLA-G†	*p*-value
Gender^**¶**^, n (%)			
Male	119 (68.8)	13.8 [13.7]	0.026
Female	54 (31.2)	6.44 [12.5]	
Age at diagnosis (years)			
Mean ± SD	59.3 ± 12.9		
Age group^**¶**^, n (%)			
≤ 60 years	87 (50.3)	6.56 [12.6]	0.168
> 60 years	86 (49.7)	9.36 [15.9]	
Type of pathology^**§**^, n (%)			
Intestinal adenocarcinoma	101(58.4)	6.82 [13.7]	0.419
Signet ring cell adenocarcinoma	45 (26.0)	8.04 [15.0]	
Other	27 (15.6)	12.5 [13.6]	
Differentiation degree^**§**^, n (%)			
Well-differentiated	15 (8.70)	9.68 [10.6]	0.607
Moderately differentiated	54 (31.2)	6.40 [17.3]	
Poorly differentiated	69 (39.9)	7.20 [11.0]	
No data	35 (20.2)	12.5 [12.8]	
Tumor location^**§**^, n (%)			
Pyloric Antrum	49 (28.3)	8.91 [17.4]	0.227
Cardias	41 (23.7)	6.36 [11.2]	
Body	73 (42.2)	9.33 [14.4]	
No data	10 (5.80)	13.2 [7.72]	
Tumor stage^**§**^, n (%)			
In situ	1 (0.60)	127**	
I	14 (8.10)	3.35 [6.13]	0.082
II	29 (16.8)	7.85 [16.7]	
III	47 (27.2)	6.89 [13.5]	
IV	51 (29.4)	11.2 [13.2]	
No data	31 (17.9)	12.0 [12.4]	
Relapse^**¶**^, n (%)			
Yes	53 (30.6)	6.90 [14.2]	0.876
No	120 (69.4)	9.06 [14.2]	
Vital state^¶^, n (%)			
Dead	75 (43.4)	9.33 [13.9]	0.361
Alive	98 (56.6)	8.18 [13.8]	

*SD* Standard deviation.

^†^Expression level in median [interquartile range].

^¶^Wilcoxon signed rank testc.

^§^Kruskal–Wallis test.

**Only one patient presented stage in situ.

**Table 2:** Demographic and clinical characteristics in patients diagnosed with benign gastric pathologies.

Characteristic	N = 307
Gender, n (%)	
Male	134 (43.6)
Female	173 (56.4)
Age at diagnosis (years)	
Mean ± SD	50.6 ± 15.9
Institution, n (%)	
Hospital Universitario de la Samaritana	142 (46.3)
Centro Policlínico del Olaya	165 (53.7)
Type of pathology, n (%)	
Chronic gastritis	265 (86.3)
Peptic ulcer	8 (2.61)
Polyps	6 (1.95)
Others (stenosis, esophagitis, and colitis)	28 (9.12)

*SD* Standard deviation.

The most frequent type of malignant pathology was intestinal adenocarcinoma 101 (58.4%), followed by signet ring cell adenocarcinoma (26.0%). The degree of differentiation was poorly differentiated in 39.9% of the patients. Most of the patients were in advanced stages III and IV (27.2% and 29.4% respectively). The 30.6% of GC patients presented relapse of the disease and the 43.4% died at the end of follow-up. Regarding benign gastric pathologies, chronic gastritis was the most frequent (86.3%) (Table 2).

### sHLA-g expression levels

**Figure 1:**
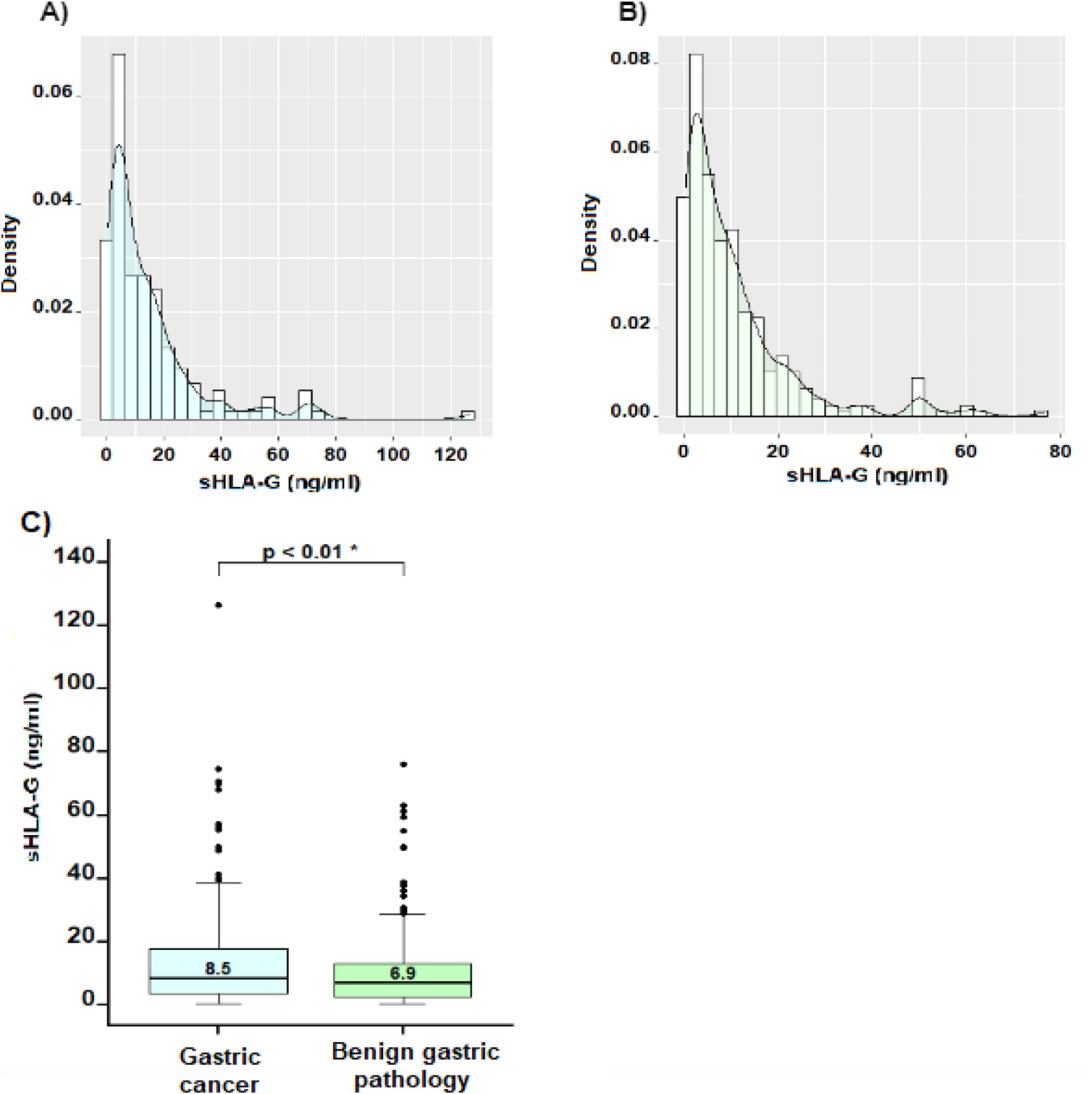
Expression levels of HLA-G in patients with gastric cancer (n = 173) and benign gastric pathology (n = 307) are shown. (**A**) and (**B**) Density plots illustrate the asymmetric distribution of sHLA-G expression levels, indicating that most observations were concentrated between 0 and 30 ng/ml. (**C**) Comparison between the groups of patients with malignant and benign pathology revealed significantly higher levels in the gastric cancer group compared to the benign pathology group (*p*-value < 0.05).

**Figure 2:**
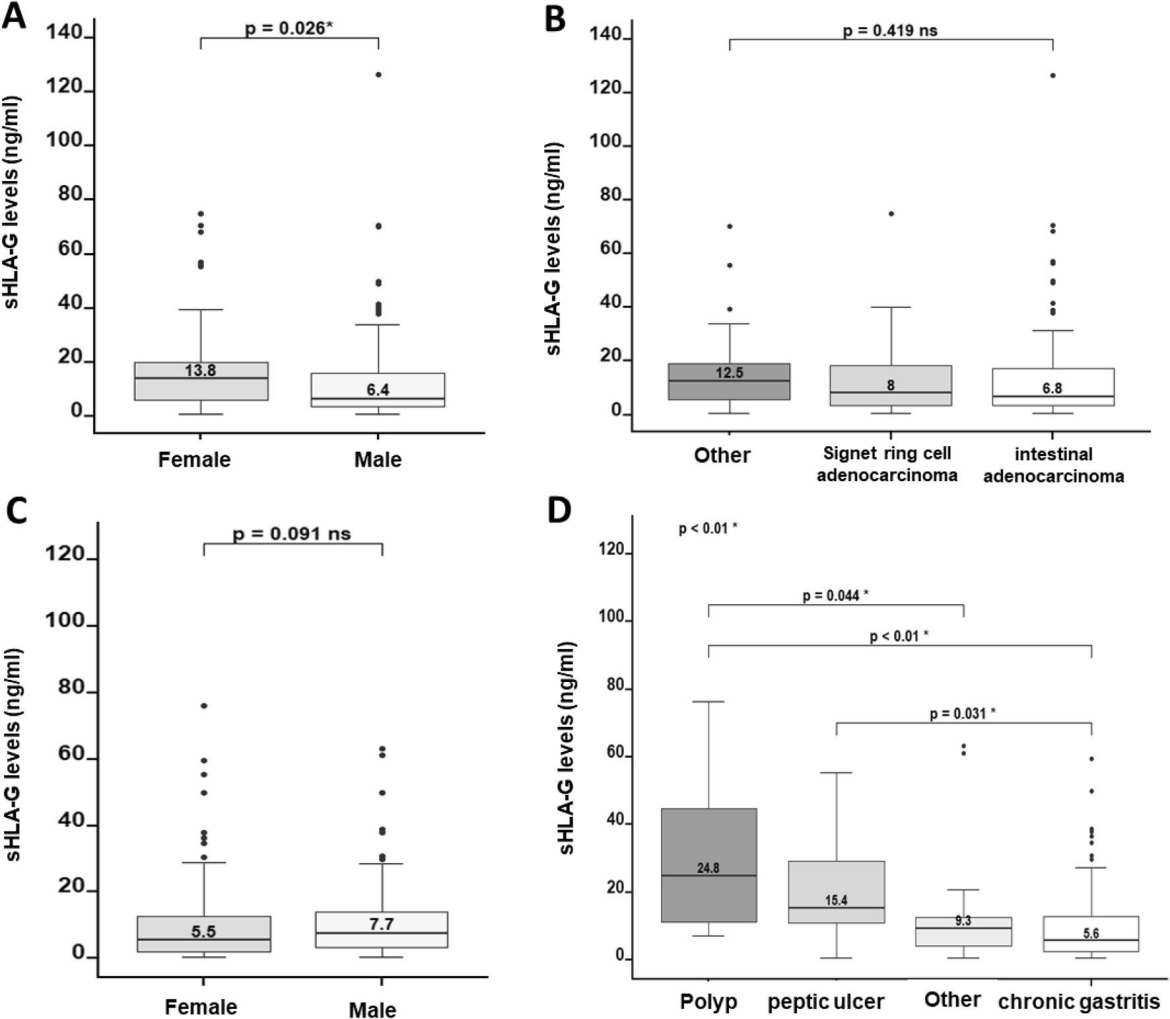
sHLA-G expression levels, by sex and type of pathology. (**A**) Women exhibited significantly higher sHLA-G expression levels compared to men. (**B**) No significant differences in sHLA-G expression levels were found when considering the different types of gastric cancer histology. (**C**) No significant differences were found in sHLA-G expression levels between men and women with benign gastric pathologies. (**D**) Within the benign gastric pathologies group, significant differences in sHLA-G expression levels were observed when comparing polyp to other types of benign gastric pathologies: polyp to chronic gastritis, and peptic ulcer to chronic gastritis. To assess the significance of these observations, quantitative variables were analyzed using the Mann–Whitney U and Kruskal–Wallis tests, while categorical comparisons were assessed using Pearson's chi-square and Fisher’s exact tests.

In both groups, the sHLA-G expression levels showed an asymmetric distributional behavior (Fig. 1A,B), with a concentration between 0 and 30 ng/ml of sHLA-G in plasma. GC patients showed significantly higher levels of sHLA-G in plasma compared with those of the benign gastric pathologies group (*p*-value < 0.01) (Fig. 1C). The median expression levels of sHLA-G in GC patients were 8.50 ng/ml (0.53–127.0 ng/ml), while in benign pathology patients was 6.92 ng/ml (0.53–76.3 ng/ml). Although among the group of GC patients, sHLA-G expression levels in women were significantly higher than in men (*p*-value = 0.026) (Fig. 2A), in the case of patients with benign pathologies, there were no significant differences in the sHLA-G expression levels between men and women (Fig. 2C). When we compared the sHLA-G expression levels between the distinct types of malignant pathologies, no significant differences were found (*p*-value = 0.419). However, the highest sHLA-G expression levels were found in the group “others” that corresponds to gastrointestinal stromal tumors (GIST) (12.5 ng/ml) (Fig. 2B). When we compared the expression levels of sHLA-G between the distinct types of benign gastric pathologies, sHLA-G expression levels were significantly different (p-value < 0.01). These differences were found between polyps and others (24.8 ng/ml vs 9.30 ng/ml, *p*-value = 0.044), polyps and chronic gastritis (24.8 ng/ml vs 5.60 ng/ml, *p*-value < 0.01) and between peptic ulcer and chronic gastritis (15.4 ng/ml vs 5.60 ng/ml, *p*-value < 0.01) Fig. 2D)**.**

### sHLA-Gev confirmation assays

We propose an initial verification of the tumor cells capability to produce HLA-G positive exosomes, in addition to release free soluble HLA-G into the extracellular space contributing to the overall expression and functional roles of HLA-G in the tumor microenvironment to delve deeper into the role of HLA-G in transmitting inhibitory signals to effector cells or activating specific signaling pathways upon fusion with the target cell membrane.

**Figure 3:**
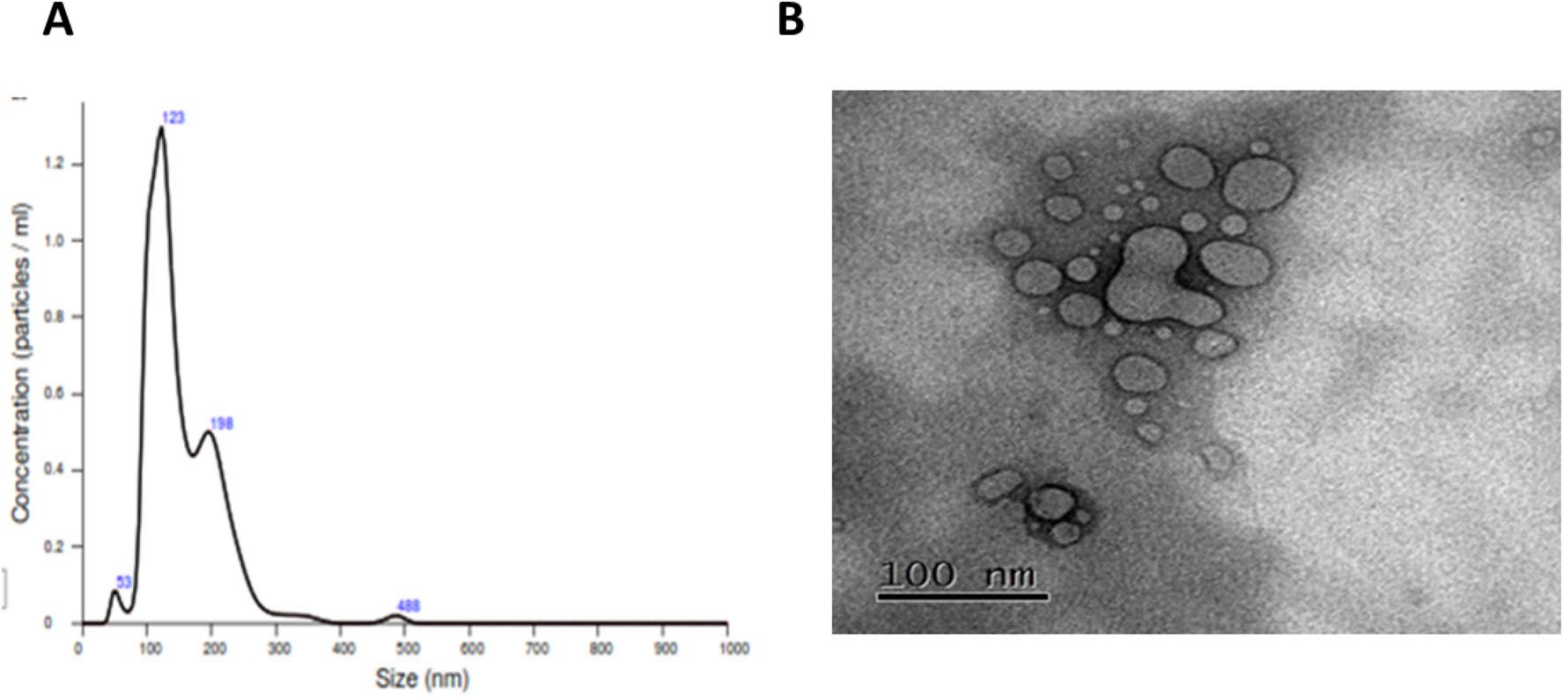
Size of the exosomes isolated from fractions 6/7 to 14 obtained by molecular exclusion chromatography of the plasma of patients with GC (n = 25). (**A**) The nanoparticle tracking analysis (NTA) screenshot shows a range of particle sizes observed between 50 and 123 nm, which corresponds to the reported size range of exosomes. (**B**) The transmission electron microscopy (TEM) screenshot displays microvesicles isolated from the plasma of gastric cancer patients, with sizes ranging from 100 nm.

Exosomes isolated from plasma of GC patients with high sHLA-G expression levels in plasma were confirmed by Nanoparticle Tracking Analysis (NTA) carried out with a pool of 5 samples per study group. We included cases of benign pathologies and GC in stages 1, 2, 3 and 4, for 25 samples. This method allows us to visualize and measure nanoparticles in suspension, based on the analysis of the Brownian movement through light scattering. Exosomes were obtained from the 6–7th to 14th fraction of each sample extracted through molecular exclusion chromatography and the presence of isolated particles ranging between 100 and 200 nm, which corresponds to the size of exosomes reported in the literature, was demonstrated by NTA (Fig. 3A).

In addition to NTA, we performed a TEM analysis on a pool of 5 samples from patients at various stages of the disease, to verify the shape and size of the particles evaluated in each sample. TEM analysis showed spherical structures with a size varying between 50 and 110 nm (Fig. 3B), consistent with previously reported characteristics of exosomes, and like the one reported for the NTA (200 nm) (Fig. 3B) which allows us to confirm the results obtained by NTA and carry out a dot blot assay to identify proteins from the tetraspanin superfamily, which are among the most abundant membrane proteins of EV. A total of five samples were analyzed using dot blot technique with monoclonal antibodies targeting CD9, CD63, TSG-101 and HLA-G proteins.

**Figure 4:**
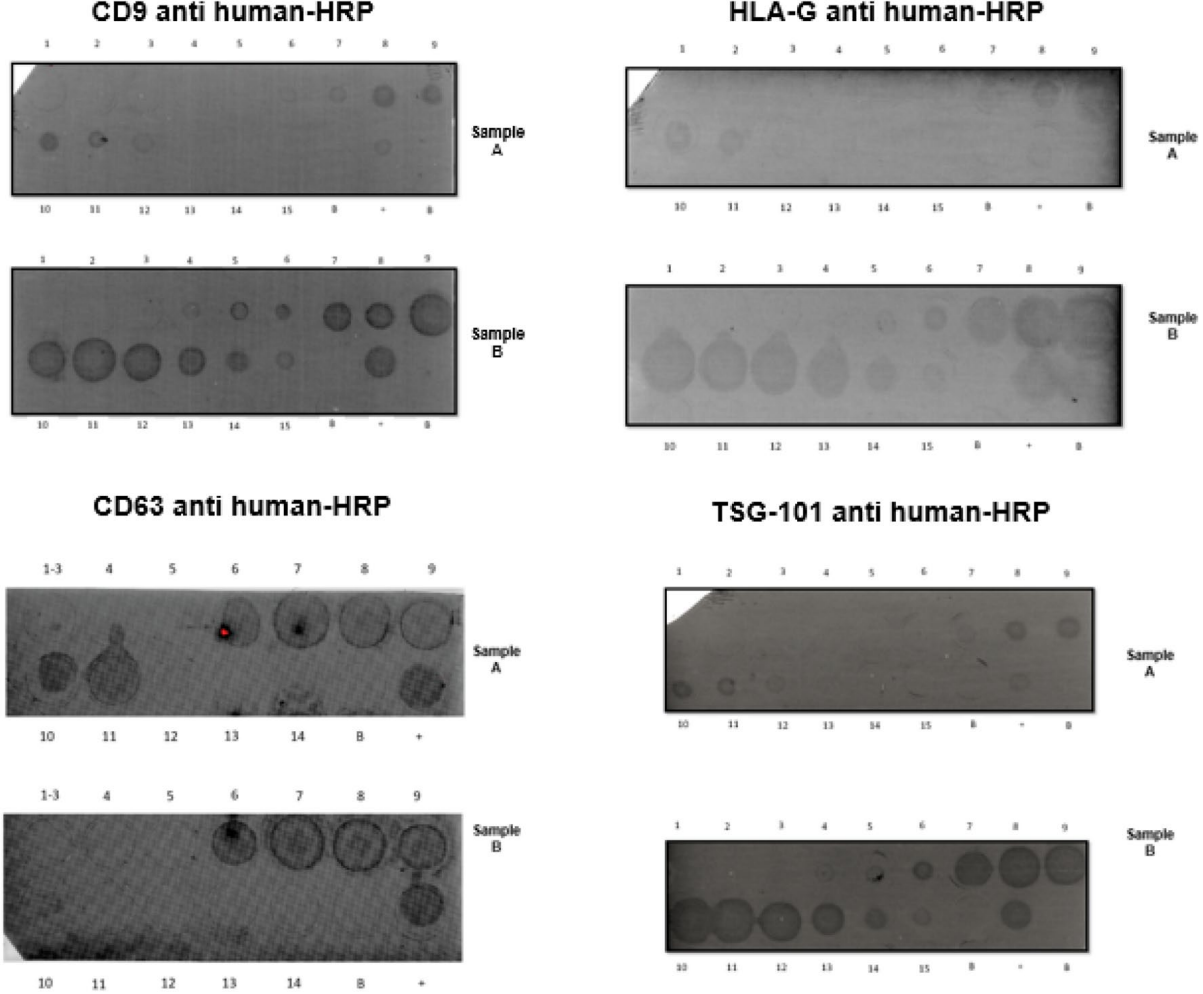
Dot blot for Isolated exosomes by molecular exclusion chromatography was performed on 5 plasma samples of patients with gastric cancer. Each fraction obtained was probed for Tetraspanins CD9, CD63, TSG-101, and for HLA-G. The expression of these proteins can be observed from Fraction 6 to 12, indicating the presence of exosomes as reported for this technique. dot blot.

Positive results for tetraspanins CD9, CD63 and TSG-101 were found from the 6–7th to 14th fractions of each sample extracted and HLA-G positive exosomes were observed in the same fractions (Fig. 4). Regarding the flow cytometry, we found HLA-G expression in all exosome samples analyzed (Data not shown). To confirm these results, we perform a negative control by adding 20 µl of sterile filtered 1X PBS instead of the 20 µl of exosome concentrate sample. The result of this flow cytometry was positive for HLA-G expression.

### Survival analysis

The median follow-up for the group of GC patients was 6.4 months, with a minimum of 0.03 and a maximum of 22.1 months. To evaluate the sHLA-G level impact on survival, a cut-off point of 39.95 ng/ml was estimated by hierarchical cluster analysis. Samples with sHLA-G levels ≤ 39.95 were determined as low expression whereas samples with sHLA-G levels > 39.95 were considered as high expression. Considering the above, n = 15 patients were classified as having high expression of sHLA-G. During the follow-up period, 56 patients (32.9%) relapsed, 74 (43.5%) died of the disease, and 1 (0.58%) died for other causes.

**Figure 5:**
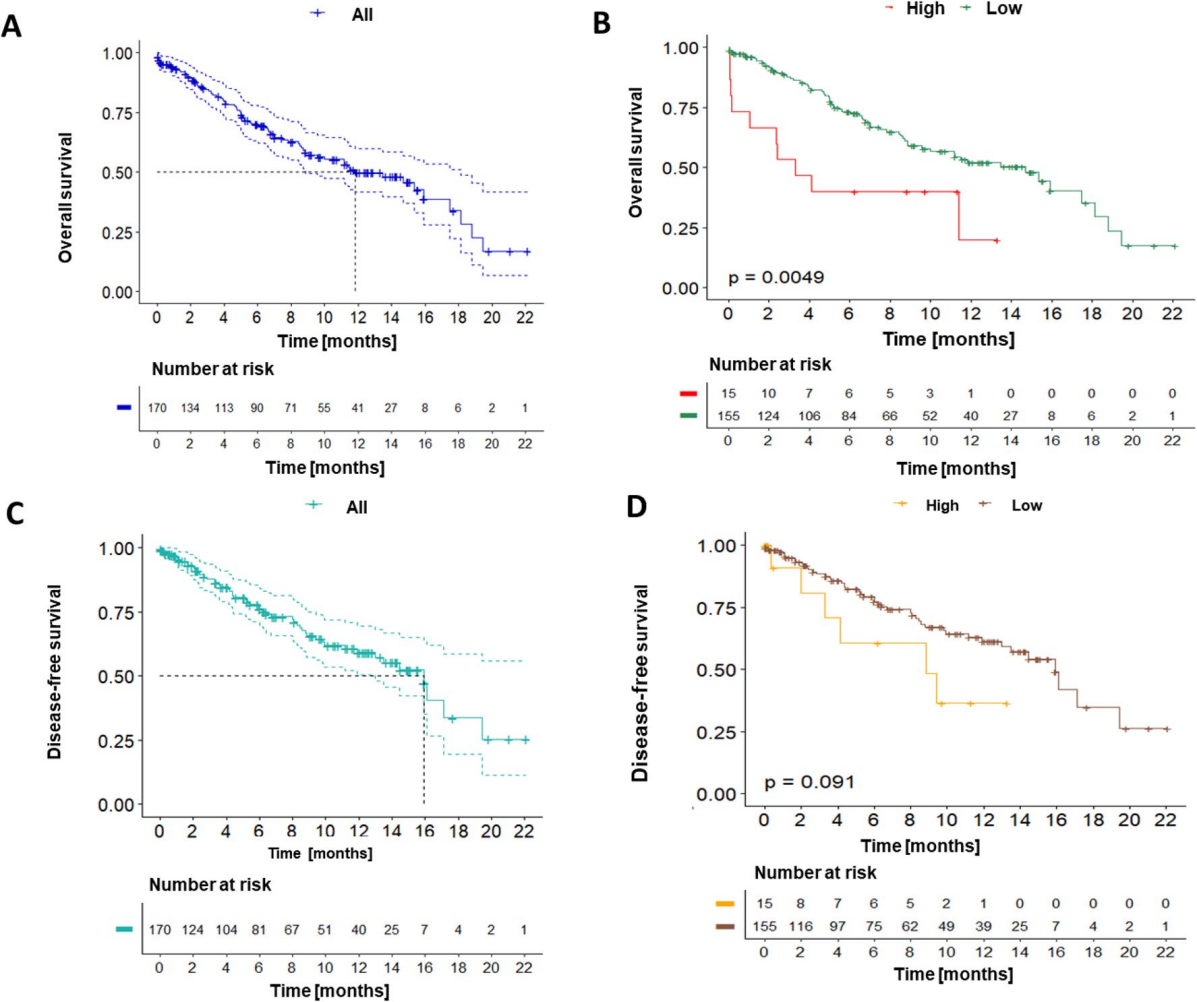
Kaplan–Meier curves were generated to analyze overall survival (OS) (Fig. 5A,B), and disease-free survival (DFS) in the entire cohort of gastric cancer patients as well as stratified by sHLA-G expression levels (Fig. 5C,D). (**A**) The 12-month OS for the entire cohort was 49.9% with a median survival of 11.8 months. (**B**) Patients with high sHLA-G expression had a significantly lower 12-month OS compared to those with low expression. (**C**) The 12-month DFS for the entire cohort was 59.2% with a median survival of 15.9 months. (**D**) Patients with High HLA-G expression had a lower 12-month DFS those with low expression group (*p*-value > 0.05)., hierarchical cluster analysis using Ward's method of minimum variance was performed to determine that the cut-off point for sHLA-G levels was 39.95.

Overall survival (OS) at 12 months for the entire cohort of patients with GC was 49.9% 95% CI (Confidence Interval): [41.6–59.7], with a median survival of 11.8 months [8.88–18.1] (Fig. 5A). Patients with high sHLA-G-expressing tumors showed a lower 12-month OS (20.0% vs. 52.1%, *p*-value < 0.01) (Fig. 5B). DFS for the entire cohort was 59.2% 95% CI [50.3–69.6] with a median survival of 15.9 months 95% CI [13.0–19.3] (Fig. 5C). DFS at 12 months was 36.4% in the high expression group and 61.4% in the low expression group (*p*-value > 0.05) (Fig. 5D).

According to unadjusted Cox proportional hazards regression analysis, sHLA-G expression levels and tumor stage were found to be univariately associated with OS (*p*-value < 0.05) (Table 3), and in DFS, univariate association were found only between sHLA-G expression levels and the stage (*p*-value < 0.05). In this cohort, no statistically significant associations were found between OS and DFS with other clinicopathological variables such as age, sex, type of pathology, and degree of differentiation. Taking the above results into account, an adjusted analysis was performed in which sHLA-G expression levels (high vs low) and tumor stage (early vs advanced), were included as covariates. This analysis showed that the effect of the sHLA-G expression levels on the OS after adjusting for clinical stage, was significant.

**Table 3:** Cox regression analysis.

Factor	Overall survival (OS)				Disease-free survival (DFS)			
	Unadjusted		Adjusted		Unadjusted		Adjusted	
	HR [CI 95%]	*p*-value	HR [CI 95%]	*p*-value	HR [CI 95%]	*p*-value	HR [CI 95%]	*p*-value
sHLA-G								
Low	1.00 (Ref.)	–	1.00 (Ref.)	–	1.00 (Ref.)	–	1.00 (Ref.)	–
High	2.39 [1.07–5.34]	**0.034†**	2.26 [1.01–5.07]	**0.041†**	1.99 [0.78–5.09]	0.200	1.85 [0.72–4.75]	0.200
Tumor stage								
Early	1.00 (Ref.)	–	1.00 (Ref.)	–	1.00 (Ref.)	–	1.00 (Ref.)	–
Advanced	3.04 [1.48–6.23]	** < 0.01†**	2.99 [1.46–6.13]	** < 0.01†**	6.27 [2.23–17.6]	** < 0.01†**	6.20 [2.21–17.4]	** < 0.01†**

*HR* hazard ratio, *CI* confidence interval, *sHLA-G* Human leukocyte antigen G level.

^†^*p*-value < 0.05.

Significant values are in [bold].

The adjustment result showed an HR = 2.26 (95% CI [1.01–5.07], *p*-value < 0.05) for the expression levels of sHLA-G and an HR of 2.99 95% CI [1.46–6.13], *p*-value < 0.05) for the clinical stage. Therefore, our results suggest that gastric cancer patients with high sHLA-G expression levels in plasma have two times more risk of death compared with patients with low sHLA-G levels, based on the adjustment made for the clinical stage of the disease.

## Discussion

The non-classical human leukocyte antigen (HLA) class I protein G (HLA-G) is an immune checkpoint molecule which exhibits limited tissue distribution. It is naturally expressed during pregnancy, playing a critical role in mediating immune tolerance at the maternal–fetal interface. Recent studies demonstrated that an ectopic up-regulation of HLA-G in cancer cells may favor their escape from antitumor immune responses^9,21^. In this study, we propose the evaluation of sHLA-G levels in plasma samples from patients with GC and benign gastric pathology, using an ELISA test, to determine if sHLA-G expression levels may be associated with clinicopathological variables. With this work, we sought to determine this molecule's clinical utility as a screening test for the early detection of GC.

The GC patients showed significantly higher levels of HLA-G compared to those of the benign gastric pathology group (Fig. 1). Our results agree with those obtained in previous studies. Pan et al. measured the plasma level of sHLA-G by ELISA, in GC patients, benign gastric pathologies patients and normal controls. They also determined serum levels of tumor markers such as AFP (Alpha Feto Protein), CEA (carcinoembryonic antigen), CA125, CA19-9 and CA72-4 to compare with sHLA-G plasma levels. As in our study, patients with GC showed higher HLA-G levels with respect to patients with benign gastric disease, highlighting the importance of sHLA-G as a potential biomarker for GC diagnosis. They also suggest the detection of sHLAG in combination with tumor biomarkers (CA125 + CA19-9 + sHLA-G or CA125 + CA724 + sHLA-G) for GC discrimination^12^. In addition, Farjadian et al. evaluate the HLA-G expression in tumor tissues and sHLA-G plasma levels in patients with gastrointestinal cancer and found plasma levels of sHLA-G significantly higher in patients with gastrointestinal cancer than in healthy controls^14^, which is correlated with our findings. Increased plasma levels of sHLA-G have also been described in a plethora of solid tumors such as cutaneous melanoma, glioblastoma multiforme, esophageal squamous cell carcinoma, non-small cell lung cancer, ovary^10^ and colorectal cancer^22^, among others, in correlation with an adverse prognosis and tumor immune evasion, since HLA-G has inhibitory effects on all immune cells, and by inducing Treg cells^14–23^.

In GC patients, sHLA-G expression levels were significantly higher in women compared to men, in opposition to the benign pathology group where no gender-related differences were observed (Fig. 2A,C). We think that this could be related to the fact that progesterone induces HLA-G expression in vitro^24^, or to other pathologies that could be associated with elevated HLA-G levels as in advanced endometriosis^25^; however, we did not find sHLA-G overexpression in women with benign gastric pathologies and since no additional clinical information was requested, it is not possible to associate our HLA-G results with this information.

On the other hand, Murdaca et al. reported that they found no significant correlation between the expression of HLA-G and other clinicopathological variables such as sex, age, stage, grade, histotype^26^, but Yan et al. reported a worse OS in patients whose tumors had high HLA-G expression and a poor clinical outcome among women with GC expressing HLA-G and ILT-4. They suggest that these tumor phenotypes are a sex-dependent prognostic factor in GC patients. Although there are few studies on gender differences in gastric cancer and it is not possible to establish that the expression level of sHLA-G has a direct relationship linked to gender^13^, a retrospective study of Han et found clinical differences of gastric cancer between women and men that should be considered in terms of personalized medicine. They reported that early gastric cancer is significantly higher in women than in men and that undifferentiated cancers are more frequent in women, but other characteristics such as size (more than 40 mm), location, TNM stage, presence of lymph node metastases, and presence of lymph vascular invasion not present significant differences^27^.

Significant differences were observed in the expression levels of sHLA-G between benign pathologies. In our study, we found the highest levels of sHLA-G expression in patients with polyps, whose differential expression was significant when compared with the group of "others'' (stenosis, esophagitis, and colitis) (*p*-value = 0.044) and with the group of chronic gastritis (*p*-value < 0.01). Unlike colonic polyps, whose malignant potential has been demonstrated, gastric polyps have little malignant potential depending on their histologic type, and the most common neoplastic polyp in the antrum portion of the stomach are adenomatous polyps^28^. Considering that atrophic gastritis is central to gastric carcinogenesis and that hyperplastic and adenomatous polyps often develop in atrophic mucosa; it is possible that gastric polyps may also give rise to cancer. Moreover, it seems that hypergastrinemia or hypo acidity may be the carcinogenic factor in common between gastric polyps and gastric cancer development^29^ since it has been shown that patients with hyperplastic and adenomatous gastric polyps may have an increased risk of gastric cancer^30,31^. The sHLA-G over expression in patients with polyps could be explained by the fact that HLA-G is crucial for tumor immune evasion and malignant transformation^32^.

These results suggest that sHLA-G expression levels could be useful to discriminate between cancer precursor diseases and benign pathologies, and that those hyperplastic and adenomatous gastric polyps should be subjected to continued surveillance to ensure that supervening carcinoma be detected at the earliest possible phase. However, we did not find studies documenting the sHLA-G expression in patients with benign gastric pathologies and more studies are necessary to verify that the early diagnosis of cancer should focus on the detection of preneoplastic lesions that are at risk of progressing to cancer in situ.

By the other hand, a previous study carried out by Costa Ferreira et al. to evaluate intestinal mHLA-G expression and soluble HLA-G (sHLA-G) levels in Crohn's disease (CD) and ulcerative colitis (UC) patients, showed that these inflammatory gastric diseases present increased levels of sHLA-G compared to controls. They also report an increased HLA-G expression in inflammatory cells associated with increased sHLA-G levels in both, CD and UC patients, however, HLA-G positive infiltrating cells was greater in UC than in CD samples, suggesting that the increased HLA-G positive inflammatory cells may increase the total plasma sHLA-G levels, (sHLA-G plus HLA-Gev)^33^.

This over-expression of HLA-G may reflect ongoing host strategies to suppress chronic inflammation^33^. Our results, in relation with the sHLA-G expression levels in benign gastric pathologies suggest that ectopic HLA-G expression in benign gastric pathologies could be useful to discriminate a precancerous pathology from other benign diseases, local and systemic mechanisms may be involved in the induction of HLA-G expression. As HLA-G expression was significantly higher in gastric polyps, that could become malignant depending on their histology and microenvironment, and chronic inflammation is a factor that favors tumor development, it could be hypothesized that both sHLA-G in gastric polyps may be participating regulating the anti-inflammatory response in the gastric microenvironment, to favor carcinogenesis in this group of patients. Additionally, a significantly higher sHLA-G expression was found in peptic ulcer than in chronic gastritis.

It has been shown that gastric ulcer (GU) is associated with a significantly increased risk of developing gastric cancer (GC)^34^, and that its most common cause is Helicobacter pylori infection. Although Oliveira Souza et al., evaluated HLA-G expression in the gastric microenvironment with H. pylori infection by immunohistochemistry and detected HLA-G expression in 43 of 54 specimens associated with milder H. pylori colonization, inflammatory activity, and localization of the bacteria in the gastric antrum^35^. By the other hand, Genre et al., suggests that the HLA-G 14-bp Ins/Del polymorphism allele could confer a greater risk of developing H. pylori infection, in addition to its association with inflammatory and autoimmune diseases as well as some viral and parasitic infections^36^. In our study, it was not possible to collect all data related to H pylori infection and thus, we cannot know if the sHLA-G overexpression that we report here is related to the infection or the tumor and it is important to design studies that make it possible to differentiate between benign pathologies that are precursors of gastric cancer and other benign gastric pathologies to determine if the high expression of sHLA-G may be a risk factor to develop gastric cancer.

A lower 12-month OS and DFS was observed in the patients with high sHLA-G expression levels compared with patients with low sHLA-G expression levels (*p*-value > 0.05). Wan et al.^37^ which explored the HLA-G expression in GC tissues obtained from 49 patients with GC by immunohistochemistry and western blot observed HLA-G expression in most of the GC tissues, correlated with poor prognosis of the disease and negatively associated with the number of tumor-infiltrating NK (Natural Killer) cells. Other studies also report the HLA-G expression associated with poor prognosis: Chen et al. found a relative shorter OS in GC patients with high HLA-G expression and a high percentage of GC cells HLA-G + and ILT-4 + are associated with a poor clinical outcome among GC patients^15^; Murdaca et al. found that HLA-G expression is associated with poor survival in patients with stage III GC; Yie et al. reported that patients with HLA-G positive tumors had a significantly shorter survival time than those patients with HLA-G negative tumors^26^ and Tuncel et al., observed that patients with HLA-G-positive primary GC had a significantly poorer prognosis than patients with HLA-G-negative tumors and that HLA-G expression and stage were independent unfavorable factors for patient survival^11^. This reduction in OS and DFS is due in part to the fact that expression of immune checkpoint proteins is one of the main evasion mechanisms used by tumors to evade innate and adaptive antitumor immune responses, promoting disease progression^38^.

The relationship between high expression levels of sHLA-G and the decrease in OS and DFS observed in our results can be explained by the immune suppression induced by the interaction between tumor HLA-G and ILT-2/-4 expressed on the immune cells surface. This interaction can inhibit T cell proliferation and B cell immunoglobulin production^39,40^, can modify T and NK cells cytotoxicity, and inhibit DC maturation^41,42^ and promotes the proliferation of Myeloid Derived Suppressor cells (MDSCs), regulatory T cells (Tregs), tolerant DC-10 cells, invariant natural killer T cells (iNKT), and the accumulation of M2-type macrophages^43,44^, and damping down chemokine receptors expression in B, T, and NK cells infiltrating the tumor, favoring the progression of the disease^45^.

In this study, exosomes isolated from plasma of GC patients with high sHLA-G expression levels in plasma were obtained and confirmed by NTA, transmission electron microscopy and dot blot of the plasmas. HLA-G positive exosomes (sHLA-Gev) were demonstrated among the 7th and 14th chromatographic fractions using antibodies against three commonly used exosome markers: the tetraspanin molecules CD9, CD63 and TSG101. HLA-Gev were also confirmed between the 7th and 14th chromatographic fractions. Subsequently, we performed flow cytometry to check for HLA-G positive exosomes and we found HLA-G expression in all exosome samples analyzed However, when we perform a negative control, it was again positive for HLA-G expression. This result suggests that the latex beads used to attach the exosomes were sequestering the anti-HLA-G antibody. These latex beads are widely recommended for performing exosome flow cytometry, but nonspecific binding is the most frequent problem when working with them, since they are hydrophobic and in biological systems, most nonspecific binding problems are the result of hydrophobic interactions. Although we block with agents such as BSA, egg albumin and complete serum, we were unable to design a satisfactory negative control and thus, we did not take these results into account because we consider that with the previous analyzes and the dot blot results it is enough to confirm the presence of HLA-G positive exosomes. We assume that the HLA-G molecule found on exosomes corresponds to the membrane-bound isoform. We use the antibody clone 4H84, which specifically recognizes HLA-G molecule associated with beta-2 microglobulin. However, we cannot definitively speculate on whether the HLA-G molecules found in the exosomes are exclusively monomeric or dimeric, it is known that the dimerization of HLA-G monomers can occur in both membrane-anchored and soluble isoforms so, further investigations are needed to determine the precise molecular composition of the HLA-G molecules within the exosomes.

Our findings have the potential to contribute to advancements in therapeutic applications involving exosomes. The expression of HLA-G in exosomes may provide valuable insights into the role of HLA-G and exosomes in the development of gastric cancer. Additionally, studying HLA-G expression in exosomes could help us identify benign lesions that have the potential to progress into malignant ones. These implications highlight the importance of further research in this area.

## Methods

### Patients

480 patients with GC and benign gastric pathology were en-rolled in this study. 173 of them, attending the gastroenterology unit at the Instituto Nacional de Cancerología (INC), from October 2018 to February 2022 with a primary diagnosis of GC and 307 as a control group with benign gastric pathology from the Centro Policlínico del Olaya and the Hospital Universitario de la Samaritana in Bogotá, Colombia. The Ethics Committee of the participating institutions approved the protocol for the collection of human plasma samples. Written informed consent was obtained from all patients according to the guidelines of the institutional review board. The inclusion criteria for GC patients were desire to participate in the study and primary diagnosis of GC, and for benign gastric disease patients were to have a benign gastric disease diagnosis confirmed by endoscopy-biopsy not older than 1 year. Exclusion criteria were had under-gone any cancer therapy before, previous neoplastic disease, active viral infection, autoimmune disease, transplantation, or pregnancy. Clinicopathological information was obtained from clinical records review, and written informed consent was obtained from all patients according to the guidelines of the institutional review board.

### Sample collection

Peripheral blood samples were collected in a 4 ml EDTA tube and plasma was isolated by centrifugation at 1500 rpm for 10 min and subsequently stored at -80 °C until further analysis.

### Detection of sHLA-G plasma levels

Plasma levels of HLA-G were quantified in 173 patients with GC and 307 with benign gastric pathology, with the Elabscience Human MHCG/HLA-G ELISA Kit (Catalog No. E-EL-H1663) whose detection limit is 0.38 ng/ml. The kit was used according to the manufacturer’s instructions. The sHLA-G concentration in each sample was calculated from a standard curve constructed in each ELISA plate by plotting the absorbance against the corresponding known calibration concentrations ranging from 0.63 to 40 ng/ml. OD was measured at 450 ± 2 nm.

### Exosomes isolation from plasma samples

Neo-ectopic expression of HLA-G molecules on the tumor cells surface or released as soluble forms, free or as part of exosomes, plays a crucial role in cancer progression. sHLA-G levels have been associated with cancer development but its diagnostic or prognostic value in terms of progression and survival, remains uncertain. It is unclear whether tumor cells produce HLA-G+ exosomes or secrete free sHLA-G, and whether both components contribute to immune evasion by tumor cells. In this study, we investigate whether HLA-G positive exosomes can be isolated from the plasma of patients with gastric cancer (GC)^14^. Exosomes isolation was performed by centrifugation cycles of 500 gravities for 5 min followed by 2000 gravities for 10 min. Then, using Amicon centrifugal filter units Ultracel 3 K Kit, Merck, an exosome enriched plasma concentrate was obtained, which was subjected to molecular exclusion chromatography using Sigma Sepharose 2B CL columns for exosomes isolation. Elution was performed by gravity with PBS (Phosphate Buffered Saline), collecting fourteen fractions from each sample, in which exosomes are expected to be found.

### Exosome confirmation assays

Chromatographic isolated exosomes were confirmed with three methodologies that allow us to: (i) identify exosomes morphology (Transmission Electron Microscopy (TEM)), (ii) determinate their size (Nano-Tracking Analysis (NTA)) and (iii) identify exosome protein markers (dot blot), a simple and sensitive method for the detection of surface proteins in the membrane of extracellular vesicles. Dot blot assays were conducted in 5 samples to confirm the presence of exosomes in the obtained exosome fractions. Samples were chosen based on their high plasma sHLA-G levels, as determined by ELISA. Exosome markers, such as CD9, and CD63, as well as immune checkpoint HLA-G molecule was used to perform dot blot assays to verify the presence of HLA-G positive exosomes.

### Nanoparticle tracking analysis

Nanoparticle Tracking Analysis (NTA) was used in 25 exosomes samples as described by Menezes-Neto to determine nanoparticles size and concentration in a liquid suspension^17^, based on light scattering and Brownian movement^18^. We used the Nano Sight LM10 system (Malvern Technologies, Malvern, UK) and nano tracking analysis (NTA) software version NTA 3.1.

### Transmission electron microscopy

Extracellular vesicle presence was demonstrated in 5 µl of fresh exosome concentrate, obtained from a pool of 5 samples from patients at various stages of the disease, using the JEM-1400 Flash Electron Microscope and a high sensitivity sCMOS camera. Once the samples were processed, readings were taken at 80 kV.

### Dot blot

Dot blot was performed with a standard procedure on a 0.2 µm nitrocellulose membrane (Bio-Rad, 1,620,146). Using 5 µl of the extracellular vesicles concentrate to confirm exosomes isolation. A total of five samples were analyzed using dot blot technique with monoclonal antibodies targeting CD9, CD63, TSG-101 and HLA-G proteins. Exosome membrane tetraspanins were evaluated using antibodies against CD9, CD63 (Biolegend, clones H19a and H5C6 respectively) TSG-101(Invitrogen clone 4A10), CD81 (Biolegend, clone 5A6) and Anti-IgG (HRP) secondary antibody (ABCAM, ab6789), and for protein detection, Clarity Max Western ECL Substrate (Bio-Rad, 1,705,062) was added and determined by chemiluminescence in the ChemiDoc imaging system analyzer (Bio-Rad).

### Flow cytometry

Exosomes were characterized by flow cytometry to evaluate tetraspanins CD9, CD63, CD81 and membrane bound HLA-G. Exosomes obtained from plasma of patients with GC who expressed elevated levels of HLA-G by ELISA were coupled to aldehyde/sulfate latex microspheres (beads) with a diameter of 4 μm, which enables exosomes detection or characterization by labeling with specific antibodies, following the protocol proposed by Monguió et al.^19^ with some modifications. Briefly, beads stock was diluted 1:10 to generate a working bead solution, and 4 μl per sample was added into a 1.5 ml microcentrifuge tube to be analyzed. An additional tube was prepared as a negative control. 20 µl of each sample was added to the corresponding tube with beads and size exclusion chromatography (SEC) buffer was added to the negative control. Subsequently, 1 ml of Bead Coupling Buffer (BCB) was added to each tube and were incubated at room temperature overnight.

For immunolabeling, the tubes were centrifuged at 2000×*g* for 10 min and the supernatant was removed, the bead-coupled samples in BCB were resuspended and labeled with CD9, CD81, CD63, and HLA-G antibodies diluted in 50 μl of BCB to be analyzed. Samples were transferred into cytometry tubes and the cytometer reading was performed.

### Statistical analyses

Statistical analyses were performed in R Project software version 4.1.1 (R Core Team, 2021). Normality was assessed for variables of a continuous nature using the Kolmogorov–Smirnov test. Variables with normal distribution were characterized by the mean and standard deviation, otherwise, the median with an interquartile range was used. Absolute and relative frequencies described qualitative variables. Comparisons between groups for quantitative variables were performed with the Mann–Whitney U test and the Kruskal–Wallis test. Categorical comparisons between groups were calculated with Pearson’s Chi-square test and Fisher’s exact test. Overall survival (OS) was calculated from the date of biopsy to the date of death or last contact. As complete treatment information was not available, disease-free survival (DFS) was calculated from the day of sampling collection to the date of disease relapse or last contact. For OS and GDF events, the survival function was calculated using the Kaplan Meier method and the different subgroups were compared using the log-rank test. To assess the impact of sHLA-G levels on survival (high expression vs low expression), the cut-off point was determined by hierarchical cluster analysis through Ward's minimum variance method. These statistical methods were used because there is no reference value to determine when it is a high or low expression in malign or benign gastric lesions. However, In the serum of healthy people has been reported that the content of HLAG is 20 ng/ml and significantly lower compared with cancer patients^20^. The effects of possible prognostic factors on OS and DFS were investigated using the Cox proportional hazards model. The significance level used was α = 0.05.

### Institutional review board

The study was conducted in accordance with the Declaration of Helsinki and approved by the Ethics Committee of The Instituto Nacional de Cancerología (protocol C19010300-408 January 19th, 2017).” for studies involving humans.

### Informed consent

Informed consent was obtained from all subjects involved in the study and written informed consent has been obtained from the patients included in this study.

### Supplementary Information

Supplementary Information.

## Data Availability

All data generated or analyzed during this study are included in this published article as supplementary files.
